# Myasthenia Gravis-Like Syndrome Resulting From Immune Checkpoint Inhibitors in a Patient With Urothelial Carcinoma

**DOI:** 10.7759/cureus.60003

**Published:** 2024-05-09

**Authors:** Alvaro J Vivas, Umar Chaudhry, Naveen Punchayil Narayanankutty, Ramon Lopez, Jorge Lamarche

**Affiliations:** 1 General Practice, Fundacion Valle del Lili/ Universidad Icesi, Cali, COL; 2 Nephrology, University of South Florida Morsani College of Medicine, Tampa, USA; 3 Nephrology, James A. Haley Veterans' Hospital, Tampa, USA

**Keywords:** ipilimumab, myasthenia gravis, autoimmunity, urothelial carcinoma, immune-checkpoint inhibitors

## Abstract

The widespread use of immune checkpoint inhibitors (ICIs) for the treatment of various types of cancer has led to increasing reports of associated adverse effects. The use of the ipilimumab/nivolumab/sacituzumab combination is currently under study in patients with metastatic urothelial carcinoma, given their potential synergism for immunogenic cell death. Information regarding the toxicity spectrum of this combination is lacking. Here, we describe a patient with urothelial carcinoma who had a severe multisystem autoimmune-like toxicity and myasthenia gravis-like syndrome in response to the ipilimumab/nivolumab/sacituzumab combination therapy. We also briefly describe the literature regarding the association between combined immunotherapy use and systemic and neurological autoimmunity.

## Introduction

Immune checkpoint inhibitors (ICIs) are immunomodulatory antibodies that enhance anticancer immune response [1]. They target immunologic receptors on the surface of T lymphocytes [1]. Some of the primary targets include programmed cell death receptor 1 (PD-1), and cytotoxic T lymphocyte-associated antigen 4 (CTLA-4). Examples of antibodies targeting PD-1 include nivolumab, pembrolizumab, and cemiplimab. On the other hand, ipilimumab and tremelimumab target CTLA-4. During the last decade, immunotherapy has been approved for a wider range of malignancies, including different solid tumors [2]. Immune-related adverse effects (irAEs) are a mosaic of inflammatory responses that can involve different organs [3]. Rarely, immune-related neurological cases of toxicities can occur [4]. Sacituzumab govitecan is a monoclonal antibody composed of anti-trophoblast cell-surface antigen 2 (Trop-2). Several trials are exploring its activity in different malignancies (NCT04468061, NCT04448886, NCT05675579). Currently the ipilimumab/nivolumab/sacituzumab combination is under study, with promising results (NCT04863885). We describe the case of a urothelial carcinoma male patient who experienced a severe inflammatory response and myasthenia gravis-like syndrome after receiving ipilimumab plus nivolumab combined with sacituzumab.

## Case presentation

A 74-year-old patient with a past medical history of chronic kidney disease (CKD) stage three and urothelial carcinoma treated with cisplatin and radical prostatectomy 18 months prior presented to the hospital with a recurrence of his malignancy. He was cisplatin-ineligible due to decreased glomerular function rate (GFR). Therapy with ipilimumab, nivolumab, and sacituzumab was initiated. After three weeks of treatment, the patient presented with complaints of fatigue, muscle aches, nausea, double vision, and loose stools. On physical exam, he was hemodynamically stable, with bilateral diplopia and loss of visual acuity. The rest of the neurological exam was normal except for decreased bilateral hip flexor strength (+/+++++). The patient subsequently went into respiratory failure requiring intubation. Electrocardiogram showed a complete heart block, with a heart rate of 40 beats per minute. Suspicion for respiratory decompensation secondary to myasthenia gravis was considered. 

**Figure 1:**
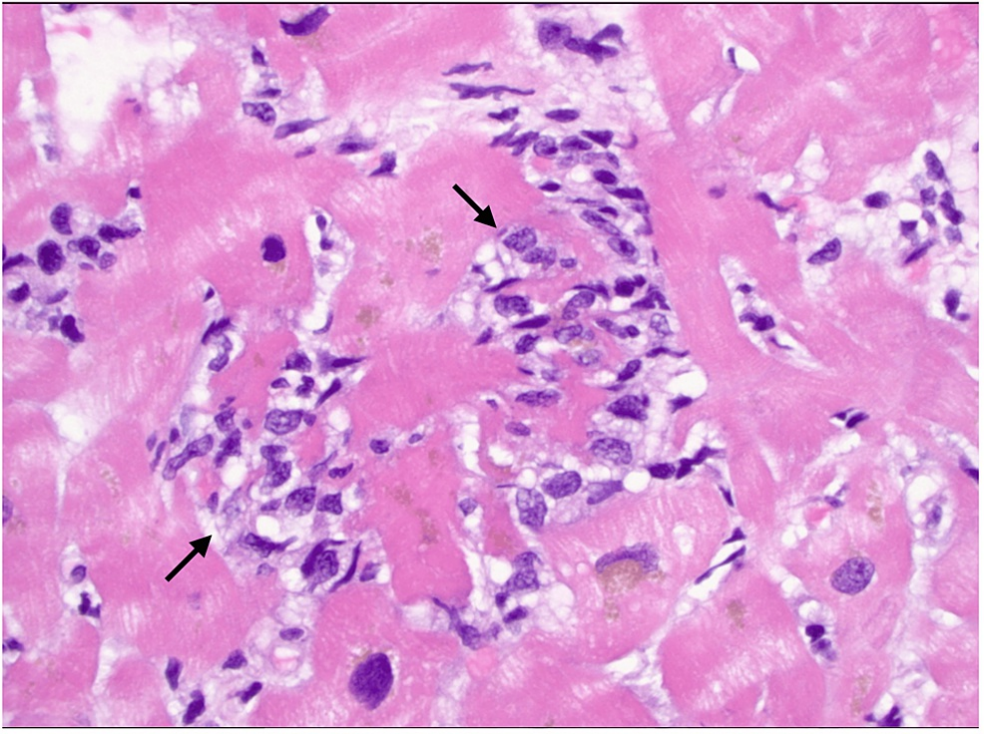
Lymphohistiocytic inflammatory infiltrates among cardiomyocytes

On blood work myositis antibodies and acetylcholine receptor-binding antibodies were negative. Other pertinent labs included elevated serum creatinine of 1.9 mg/dL (baseline was 1.5 mg/dL) and creatine kinase of 16,000 units/L. Urinalysis showed mild proteinuria and pyuria. Troponin I (high sensitivity) was 13,441 ng/L, alanine aminotransferase (ALT) was 288 units/L, and aspartate aminotransferase (AST) was 218 units/L. The patient underwent cardiac catheterization that showed multivessel coronary artery occlusion with 70% left main coronary artery ostial disease. A temporary pacer was inserted. There was a concern for myocarditis, and the patient was started on methylprednisolone, belatacept, mycophenolate mofetil, and vasopressors. Endomyocardial biopsy revealed predominantly lymphohistiocytic inflammatory infiltrates suggestive of myocarditis (Figure 1).

Intravenous immunoglobulin (IVIG) was considered as a therapeutic option. However, the patient requested to be extubated and placed on comfort measures only. The patient passed away with his family at the bedside.

## Discussion

ICIs are relatively novel therapeutic options for different malignancies, with growing evidence for solid tumors [5]. The nivolumab plus ipilimumab combination has been shown to be an effective treatment for urothelial carcinoma [6]. Systemic irAEs with the use of ipilimumab, nivolimumab, or their combination can occur. Reported phenomena include inflammatory skin reactions, diarrhea, colitis, and endocrinopathies [7]. Immune-related neurological adverse effects occur in up to 14% of patients [8]; these include Guillain-Barré syndrome, limbic encephalitis, transverse myelitis, and myasthenia gravis-like syndrome. The latter is very infrequent, with around 50 de novo cases described in the literature [9]. The incidence is higher in patients with combined CTLA-4 and PD-1 inhibition [8].

Sacituzumab govitecan is a monoclonal antibody composed of Trop-2. It has been shown to be superior (longer progression-free survival) than a single-agent chemotherapy among patients with metastatic triple-negative breast cancer [10]. Several trials are exploring its activity in different malignancies (NCT04468061, NCT04448886, NCT05675579). Currently, the ipilimumab/nivolumab/sacituzumab combination is under study, with promising results (NCT04863885). The most common adverse effects described for sacituzumab include nausea, diarrhea, fatigue, and neutropenia [11]. The evidence regarding its immune-related or neurological side effects is scarce, with some reported descriptions of induced speech disorder, muscle atrophy, hepatic failure, and myocardial infarction [12]. However, the consequences of using nivolumab plus ipilimumab combined with sacituzumab regarding adverse effects are unknown.

Here, we describe a rare case of the ipilimumab/nivolumab/sacituzumab combination-induced myasthenia gravis-like syndrome plus severe multisystem autoimmune-like toxicity. In a systematic review of the literature, 58 de novo cases of immunotherapy-induced myasthenia gravis-like syndrome were identified, of which 37% experienced concurrent myositis [9]. Our patient presented with myositis, myocarditis, and myasthenia gravis-like syndrome, a triad that has been named “3M triad.” Patients presenting with this triad have a particularly high mortality, up to 60% [13]. Despite negative myositis-specific antibodies and acetylcholine receptor antibodies, myasthenia gravis-like syndrome and myositis could not be excluded considering immunotherapy-related seronegative cases have been described [4]. Myocarditis was corroborated on histopathological findings.

In addition, our patient presented with a particularly severe multisystemic response, including acute kidney injury, elevation in aminotransferases, and cardiogenic shock. The latter was caused due to both a complete heart block and a myocardial infarction. The estimated prevalence of immunotherapy-associated cardiotoxicity is around 2%, of which 7% comprise conduction disorders. Myocarditis has the highest mortality rates of all irAEs, up to 50% [14].

Different mechanisms have been postulated regarding how ICIs induce irAEs. These include local reduction of T-reg cells, loss of peripheral tolerance, and the release of potential antigens from the tumor that resemble normal tissue (e.g., cardiac muscle) [14]. Risk factors include genetic predisposition, pre-existing autoimmune disease, duration of therapy, and combined checkpoint blockade [15]. 

Combining nivolimumab with ipilimumab is associated with higher incidence of irAEs [8]. The risk of this therapy combined with sacitizumab remains largely unknown, as sacituzumab is currently an experimental therapy. Treatment strategies for irAEs include the interruption of ICIs, use of corticosteroids, IVIG, and plasma exchange [16]. IVIG was considered for our patient; however, he elected to go for comfort care.

## Conclusions

This case describes the potential of severe adverse effects for the ipilimumab/nivolumab/sacituzumab combination treatment. Early recognition of these manifestations is important to reduce the probability of complications and mortality. Thorough discussion with the patient considering risks and benefits should always be considered.
